# Network Analysis of DSM Symptoms of Substance Use Disorders and Frequently Co-Occurring Mental Disorders in Patients with Substance Use Disorder Who Seek Treatment

**DOI:** 10.3390/jcm11102883

**Published:** 2022-05-19

**Authors:** Edith López-Toro, Casper J. H. Wolf, Rafael A. González, Wim van den Brink, Arnt Schellekens, María C. Vélez-Pastrana, on behalf of the AFP Working Collaborative Group

**Affiliations:** 1 PhD Program in Clinical Psychology, Universidad Carlos Albizu, P.O. Box 9023711, San Juan, PR 00902-3711, USA; elopez494@sju.albizu.edu; 2 Department of Psychiatry, Radboud University Medical Center, 6525 GA Nijmegen, The Netherlands; casper.wolf@radboudumc.nl (C.J.H.W.); arnt.schellekens@radboudumc.nl (A.S.); 3 Department of Cognitive Neuroscience, Donders Institute for Brain Cognition and Behaviour, Radboud University, 6525 EN Nijmegen, The Netherlands; 4 Nijmegen Institute for Scientist-Practitioners in Addiction (NISPA), 6525 HR Nijmegen, The Netherlands; 5 National Adoption and Fostering Service & National Conduct Problems Team, Michael Rutter Centre, South London and Maudsley NHS Foundation Trust, London SE5 8AZ, UK; rafael.gonzalez@slam.nhs.uk; 6 Centre for Psychiatry, Imperial College London, London W12 0NN, UK; 7 Department of Psychiatry, Amsterdam University Medical Center, Location Academic Medical Center, Meibergdreef 5, 1105 AZ Amsterdam, The Netherlands; w.vandenbrink@amc.uva.nl

**Keywords:** network analysis, substance use disorders, ADHD, comorbidity, personality disorders, borderline personality, conduct disorder, gender differences

## Abstract

Background: Substance use disorders (SUD) often co-occur with other psychiatric conditions. Research on SUD and comorbid disorders generally flows from a categorical diagnostic or dimensional latent variable perspective, where symptoms are viewed as independent indicators of an underlying disorder. In contrast, the current study took a network analysis perspective to examine the relationships between DSM symptoms of SUD, ADHD, conduct disorder (CD), depression (MDD), and borderline personality disorder (BPD). In addition, we explored possible gender differences in the network structures of these symptoms. Method: In a sample of 722 adult treatment-seeking patients with SUD from the International ADHD in Substance Use Disorders Prevalence Study (IASP) we estimated the network structure for 41 symptoms of SUD, ADHD, CD, MDD, and BPD. We described the structure of symptom networks and their characteristics for the total sample, and we compared the symptom networks for males and females. Results: Network analyses identified seven clusters of symptoms, largely corresponding with the DSM diagnostic categories. There were some connections between clusters, mainly between some hyperactivity symptoms and CD and depressive symptoms. ADHD hyperactivity was most central in the symptom network. Invariance tests revealed no significant gender differences in the structure of symptom networks. Conclusions: The current findings support the categorical DSM classification of mental disorders in treatment-seeking patients with SUD. Future network analyses should include a broader range of symptoms and prospectively explore changes in the symptoms network of patients during treatment.

## 1. Introduction

Substance use disorders (SUD) are highly prevalent and frequently comorbid with other mental disorders [1], and with physical health conditions [2]. According to national population surveys, about half of the people with a lifetime SUD will also experience other mental disorders, and vice versa [3,4]. For example, anxiety disorders [5,6] and mood disorders [5,7,8] occur in about 30% and 41% of people with SUD, respectively. A meta-analysis of studies on the prevalence of Attention Deficit Hyperactivity Disorder (ADHD) in populations with SUD showed that ADHD is present in almost a quarter of patients with SUD [9]. Finally, personality disorders (e.g., antisocial (ASP) and borderline (BPD) personality disorders) are also very prevalent in patients with SUD, with estimated odds ratios of approximately 4 for SUD coexisting with any personality disorder [1,7,10].

Overlapping and comorbid symptoms of SUD with other mental disorders present inordinate challenges for the diagnosis and treatment of these patients [4,11,12]. Moreover, patients with SUD and comorbid disorders often have symptoms that are more persistent, severe, and resistant to treatment, compared to patients with only SUD [3,13]. Patients with SUD and comorbid disorders also have poorer treatment adherence [14] and higher treatment dropout rates [3,13], which in turn negatively impact clinical outcome. A better understanding of the clinical factors underlying these comorbidities requires critical research attention.

There is some evidence of gender differences in the psychiatric comorbidities of patients with SUD. One study showed that females with SUD are significantly more likely than males to meet diagnostic criteria for a co-occurring psychiatric disorder. For instance, women with a cocaine use disorder have higher rates of comorbid psychiatric disorders, such as mood, anxiety, and psychotic disorders, than men [15]. Furthermore, men with a cannabis use disorder have higher rates of antisocial personality disorder than women, whereas women with a cannabis use disorder have higher rates of anxiety disorders and panic attacks than men [16]. A study on personality disorders in adolescent outpatients found associations between SUD and BPD, having more than one personality disorder, conduct disorder, and ADHD, but only in girls [17]. In contrast, the prevalence of ADHD in patients with SUD does not appear to vary by gender across studies [18,19], and other studies also found no differences in the comorbidities between male and female patients with SUD [20].

In an attempt to explain the extent and the nature of psychiatric comorbidity in SUD, different models have been proposed, including, (a) SUD symptoms causing other mental disorders (e.g., withdrawal evoking panic attacks), (b) SUD as a final outcome of self-medication of anxiety, depression, or ADHD symptoms (e.g., alcohol dependence caused by self-medication for social phobia), or (c) comorbid SUD and other mental disorders as a result of shared genetic and/or environmental risk factors. However, all these models are based on the traditional medical conceptualization of psychopathology, assuming the existence of a latent disorder that determines a set of observed symptoms [21]. This view has limitations, since it presents disorders as a label of a set of symptoms that share a single causal background. It proposes that a latent disease entity causes all psychopathological symptoms, and it does not consider that symptoms can be associated over and above a common cause, both within and across disorders [22].

It has been argued that network approaches provide a useful alternative way of thinking about psychopathology that may better account for the complex relationships, including comorbidity, between psychiatric disorders [21]. From a network perspective, comorbidity is seen as an intrinsic feature of mental disorders [23]. Comorbidity arises as a result of direct relationships between symptoms that are shared by different disorders, so-called bridge symptoms [24]. Network analysis techniques provide a way to analyze psychiatric conditions in their full complexity. Network analysis states that: (a) mental disorders are best characterized in terms of the interaction between different components in a psychopathology network; (b) the components in the network correspond to symptoms, such as DSM symptoms; (c) the structure of the network emerges from a pattern of direct causal connections between symptoms; and (d) mental disorders follow this network structure.

In the present study, we used network analysis to examine the interrelationships between DSM symptoms of SUD and four frequently co-occurring mental disorders (ADHD, conduct disorder (CD), major depressive disorder (MDD), and BPD) in women and men seeking SUD treatment. We applied network analysis to map the structure of symptom associations between SUD, ADHD, CD, MDD, and BPD, and we explored possible gender differences in symptom networks. The aims of the study were: (1) to examine the interrelationships between DSM symptoms of SUD, ADHD, CD, MDD, and BPD in adult men and women seeking treatment for substance misuse using network models; (2) to examine possible gender differences in the network structures of DSM symptoms for SUD, ADHD, CD, MDD, and BPD.

## 2. Materials and Methods

### 2.1. Study Design

This study was a secondary analysis of data from the *International ADHD in Substance Use Disorders Prevalence* (IASP) study, conducted by the International Collaboration on ADHD and Substance Abuse (ICASA). The IASP study was a two-stage international multi-center, cross-sectional study conducted in 11 countries [25], and it included participants from inpatient and outpatient addiction treatment centers [26]. Data for the IASP study was collected in two stages, from 2009–2011 (IASP-1) [25] and from 2015–2018 (IASP-2) [27].

### 2.2. Participants

The total sample of the original study comprised 3960 treatment-seeking adult patients with SUD (aged 18–65 years); 3558 participated in IASP-1 [25] and 402 participated in IASP-2 [27]. Participants were excluded from the IASP-1 and -2 studies if they were unable to fill out questionnaires (e.g., due to limited literacy or language skills), were unwilling to sign informed consent forms, or had severe psychiatric and/or somatic disorders requiring immediate treatment. Patients who were intoxicated or currently suffering from severe physical or mental problems were asked to join the study when their clinical condition improved. All participants in the IASP studies gave signed informed consent after receiving verbal and written information about the study.

For the current study, we included only patients with complete data on the variables of interest to our study objectives. Thus, we analyzed data from a subsample of *n* = 772 IASP-1 and -2 cases that had complete symptom-level data for SUD, ADHD, CD, MDD, and BPD. Participants who had missing data on any of the symptom variables that were needed for the current analyses were excluded. Missing data may have been due to the instrument skip patterns described below in Section 2.3.3, or because not all participants in IASP-1 completed the ADHD diagnostic interview [25]. Refer to Table 1 for a listing of symptoms representing SUD, ADHD, CD, MDD, and BPD.

### 2.3. Instruments

#### 2.3.1. Substance Use Disorders (SUD)

The Mini International Neuropsychiatric Interview (MINI)–Substance Use Module was used to collect data on DSM-IV drug and alcohol use disorders [28]. The substance abuse and dependence module was completed for five substance classes: stimulants (amphetamines, methamphetamine, methylphenidate, etc.), cocaine (inhaled, intravenous, or crack), narcotics (opioids including heroin, morphine, opium, methadone, oxycodone, meperidine, etc.), marihuana (includes hashish), and “others” (includes benzodiazepines, barbiturates, sedatives, hallucinogens, and inhalants). For our analyses, we collapsed the seven substance dependence symptoms across all five substance classes, and we calculated a binary variable indicating the presence or absence of each of the seven DSM-IV substance dependence symptoms for any substance. This produced the seven symptom variables used for SUD (S1–S7, see Table 1) in our analyses. Each symptom was coded as “present” if it was present for at least one substance, without distinguishing between substances. The four abuse symptoms were not included as variables because of missing data due to the skip patterns of the instrumentation used to collect the symptom data (MINI).

#### 2.3.2. Attention Deficit Hyperactivity Disorder (ADHD)

The Conner’s Adult ADHD Diagnostic Interview for DSM-IV (CAADID) part II [29] was used to assess ADHD inattention, hyperactivity, and impulsivity symptoms. The CAADID is a structured clinical interview that is reliable for the diagnosis of ADHD in individuals 18 years and older [30]. It was adapted to reflect DSM-5 ADHD criteria. We used nine symptoms of inattention and nine symptoms of hyperactivity/impulsivity as the variables in our network analyses (I1–I9 and H1–H9, see Table 1).

#### 2.3.3. Other Mental Disorders

Patients were also evaluated for other mental disorders common in patients with substance abuse, including major depressive disorder (MDD), borderline personality disorder (BPD), and conduct disorder (CD), using the Mini International Neuropsychiatric Interview (MINI)-plus for MDD [28], the Structured Clinical Interview for DSM-IV (SCID-II) for BPD [31], and the Kiddie-SADS Present and Lifetime Version (K-SADS-PL) for CD [32,33]. These are reliable semi-structured diagnostic interviews frequently used in research and clinical practice [28,31,33,34]. However, the K-SADS-PL contains a skip if criteria for conduct disorder (CD) before age 15 are not met, because without the presence of CD before age 15, a diagnosis of ASP cannot be reached and there is no need to assess ASP symptoms. Consequently, we did not have ASP symptoms for all study participants, and therefore we had to use the data that were available for all patients on childhood CD symptoms as a proxy for the possible presence of adult ASP symptoms. A similar situation occurred with MDD. The MINI-plus also uses skips, and for MDD there is a skip if depressed mood is not present. Consequently, we had only information on current and lifetime depressed mood for all patients, and we had to use this limited information as a proxy for all depression symptoms.

In summary, we were able to use 41 symptoms as variables in our network analyses: 18 symptoms for ADHD (H1–H9 and I1–I9), 7 symptoms for SUD (S1–S7), 9 symptoms of BPD (B1–B9), 5 symptoms of CD (C1–C5), and 2 symptoms of MDD (D1–D2), see Table 1.

### 2.4. Procedure

The methods for participant recruitment and assessment for the IASP studies are described in detail elsewhere [25,26]. In summary, a convenience sample of 3960 adults ages 18 to 65, seeking treatment for substance use problems, from Australia, Belgium, France, Hungary, the Netherlands, Norway, Spain, Sweden, Switzerland, the United States and Puerto Rico participated in the IASP studies. Data was collected through face-to-face structured interviews. Uniform procedures and assessment protocols were used across all sites [26]. All staff were trained in the assessment instruments.

The ethical aspects of the IASP studies were approved by the Institutional Review Boards of all participating institutions, and all participants provided their informed consent after receiving verbal and written information about the study. The data collection process was confidential and voluntary. The IASP studies were conducted according to the guidelines of the Declaration of Helsinki. The Institutional Review Board of Albizu University approved the current secondary data analysis study of de-identified data from IASP. Data did not include any identifying personal information.

### 2.5. Data Analysis

#### 2.5.1. Network Analyses

A network analysis was performed for 41 DSM symptoms for SUD (7), ADHD (18), BPD (9), CD (5), and MDD (2) using the IsingFit package for R [35,36]. IsingFit estimates partial correlations among a set of binary variables, in our case, DSM symptoms. Each DSM symptom was classified as either present (1), or absent (0). IsingFit uses the least absolute shrinkage and selection operator (eLASSO) method, penalizing partial correlations between symptoms to make small correlations shrink to zero [35]. The eLASSO method is based on the extended Bayesian information criterion (EBIC), which identifies relevant relationships between binary variables. In our analysis, gamma was set to 0.25 for extra regularization of the network, and the “AND” rule was applied to only identify bi-directional relations between symptoms. A detailed explanation of the Ising model can be found in [35] and its Supplementary Materials of [35].

A visual representation of the network analysis was made both for our entire sample, and for males and females separately. This network diagram consisted of ‘nodes’, representing the included variables (in our case, DSM symptoms), and ‘edges’, representing the correlation between two symptoms, while controlling for the influence all other symptoms in the network [21,37]. Green and red edges represented positive and negative associations, respectively. The thickness of the edges represented the strength of the partial correlation between two symptoms. The layout of the diagram for the entire sample was based on the Fruchter–Reingold algorithm, where all nodes repulse each other regardless of their connection, and where connected nodes attract each other. The layouts of the male and female diagrams were an average of the separate male and female layouts based on the Fruchter–Reingold algorithm. To increase the readability of the network diagram, we only included correlations higher than 0.25.

#### 2.5.2. Comparison of Symptom Networks by Gender

We used the NetworkComparisonTest (NCT) package for R [38] to statistically compare the network structures of DSM symptoms in males versus females. The NCT is a permutation-based hypothesis test suited for Gaussian and binary data. The NCT assesses the difference between two networks using several invariance measures (e.g., network structure invariance, global strength invariance, and edge invariance). The NCT method performs well with binary data, has low type-I error, and adequate power [38,39].

Statistical analyses for network analyses (estimation and comparison) were performed using R version 4.0.5. [40]. Network diagrams were made using the qgraph package in R [41]. Descriptive analyses were performed using Stata 15.

## 3. Results

### 3.1. Demographic and Clinical Characteristics

The demographic and clinical characteristics of the participants (*n* = 772) are presented in Table 2. Seventy-seven percent of the sample identified as male. Male and female participants did not differ in demographic characteristics such as age, housing, and marital status. However, there was a slightly larger proportion of Hispanic people among the male participants (*p* = 0.005); more males were employed, and more females were unable to work because of disability (*p* = 0.023).

Alcohol, cannabis, heroin, and cocaine dependence were the main reasons for the participants to seek treatment in the addiction treatment centers. Approximately half of our total sample of treatment-seeking patients with SUD also met DSM diagnostic criteria for MDD; a third met diagnostic criteria for ASP, and a quarter for BPD. ADHD was more prevalent in male than in female patients (*p* = 0.046), whereas BPD was more prevalent in female compared to male patients (*p* < 0.001). There were no significant gender differences in the prevalence of comorbid ASP, CD, or MDD.

### 3.2. Estimation of Symptom Networks

**Figure 1:**
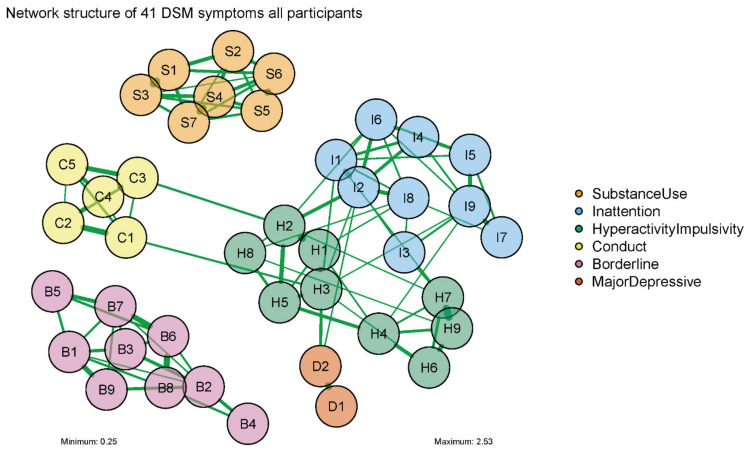
Network structure of 41 DSM symptoms of SUD, ADHD, CD, MDD, and BPD for total sample (*n* = 772) (IsingFit). Description of each symptom (node) is presented in Table 1.

Figure 1 presents the network of symptoms that we obtained for the total sample of patients with SUD. Visual inspection of Figure 1 reveals that all symptoms belonging to one DSM diagnostic category tended to cluster together. Figure 1 also shows that the symptom clusters pertaining to different diagnostic categories were only sparsely connected with each other. In particular, SUD and BPD symptoms appeared to be unconnected to the symptom clusters of other DSM diagnostic categories. In contrast, ADHD symptoms of hyperactivity/impulsivity (H1, H2, H3, and H7) were connected to symptoms belonging to the diagnostic categories of CD (C1 and C3) and MDD (D1 and D2), as well as to symptoms of inattention (I1, I2, and I3). Consequently, the hyperactivity/impulsivity symptoms were at the center of the network, and were relatively close to the symptoms of other categories outside of their own cluster. Hyperactivity/impulsivity symptoms H2 (“often has trouble remaining seated”) and H3 (“often experiences feelings of restlessness”) were connected to the CD symptoms C3 (“fights, threatened, intimidated others before age 15”) and C1 (“skipped school or stayed out before age 15”), respectively. H3 (“often experience feelings of restlessness”) was also connected to I1 (“fails to give close attention/make careless mistakes”) and to D2 (“depressed mood, lifetime”).

### 3.3. Comparison of Networks of DSM Symptoms by Gender

**Figure 2:**
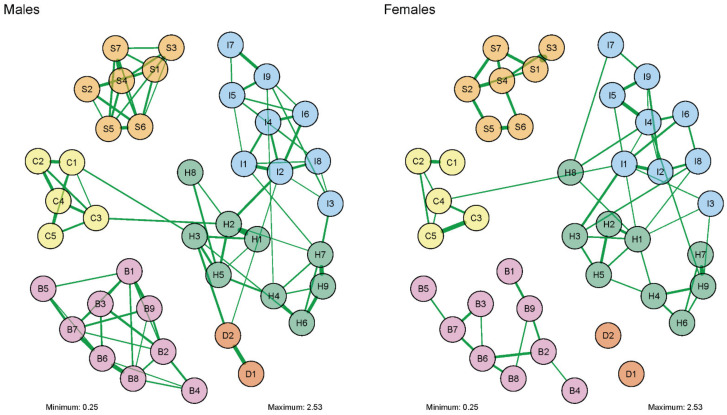
Comparison of two network structures of 41 DSM symptoms of SUD, ADHD, CD, MDD, and BPD (IsingFit). Description of each symptom (node) is presented in Table 1.

A comparison of the symptom networks of male and female patients with SUD is presented in Figure 2. Visual inspection of Figure 2 reveals that edges that were present in the males’ network of DSM symptoms seemed stronger overall than those in the females’ network, and males seemed to have a slightly denser network than females. As in the overall network, within the SUD symptom cluster there appeared to be slight differences in the number and strength of connections between symptoms in males vs. females. Nodes D1 and D2 (current and lifetime depressed mood) were not connected to other nodes in the female network of symptoms. However, the male and female symptom networks were similar in regard to the central role that ADHD symptoms of inattention and hyperactivity/impulsivity have in both. In both networks, the hyperactivity/ impulsivity symptoms are at the center of the network, and are relatively close to symptoms of other categories. Finally, in both networks, SUD and BPD symptom clusters are unconnected to other diagnostic categories.

The network structures for males and females were not significantly different. First, the network structure invariance test was non-significant (M = 1.37, *p* > 0.35; 1000 iterations). Second, the network comparison test (NCT) on invariance of global strength indicated that there was no significant difference in global strength between the two networks (*S* = 21.48, men 66.94 vs. female 45.46, *p* = 0.70). Since the test on invariance of network structure was not significant, and thus does not indicate any significant differences in individual edges between the two networks, there was no need to test edge invariance for specific edges [38]. These results suggested that the apparent differences that were visually observed were likely due to differences in sample size and/or sampling variation. Detailed results of the network analyses, including all symptom thresholds and centrality measures, can be found in in Supplementary Table S1 and Figures S1 and S2.

## 4. Discussion

The present study aimed to examine the interrelationships between symptoms of SUD and its main comorbidities ADHD, CD, MDD, and BPD, using a network analysis approach. The network that we obtained showed that all symptoms that belonged to a certain diagnostic category tended to cluster together, but to a large extent they were not clustered with symptoms from other diagnostic categories; SUD symptoms clustered together, but not with any of the symptoms of the other diagnostic categories. In contrast, some of the hyperactivity/impulsivity symptoms (ADHD symptoms) were connected with some of the CD and MDD symptoms. As a result, the hyperactivity/impulsivity symptoms had the most central position in the overall network. Like SUD symptoms, BPD symptoms were totally unconnected to symptoms of the other DSM diagnostic categories. Finally, there were no significant differences in symptom networks between males and females.

The current findings are consistent with previous work using a network approach to investigate psychiatric comorbidity [23]. For example, a study of 1059 persons from a general population sample showed that symptoms tend to cluster with one another within each disorder, but that between disorders, there are fewer, or weaker, between-symptom connections [24]. Similarly, network analyses of 120 symptoms of 12 DSM-IV disorders using data from the National Epidemiologic Survey on Alcohol and Related Conditions (NESARC) study demonstrated clustering of symptoms within the same diagnosis [42]. These findings—using network analysis—support the validity of the categorical DSM classifications as symptom clusters that are closely connected, with relatively few symptoms belonging to more than one diagnostic cluster, i.e., with relatively few bridge symptoms. In the current study, this was especially true for BPD and SUD, with no bridge symptoms at all at the applied significance level, and thus no symptoms constituting a possible symptom-specific pathway for the development of categorical comorbidity. Therefore, other causal models for the frequent comorbidity between SUD and other mental disorders should be investigated.

However, certain hyperactivity symptoms (H2 and H3) were not only connected with other hyperactivity symptoms, but also with certain CD/childhood ASP symptoms (C1 and C3), suggesting that CD/childhood ASP symptoms interact with certain ADHD symptoms—which explains at least some of the comorbidity of adult ADHD and ASP [43]. Similarly, one specific hyperactivity symptom (H3) was not only connected with other hyperactivity symptoms, but also with a lifetime depressed mood (D2). The link between hyperactivity and depression symptoms might reflect the underlying shared emotion and impulse regulation deficits [44,45,46], and thus contribute to the frequent comorbidity of MDD in patients with ADHD [5].

Some symptoms showed stronger connections with certain symptoms within their disorder category than with other symptoms, and were thus more central within those clusters. For example, both “trying to cut down or quit” (S4) within the SUD cluster, and “many sudden mood swings” (B6) within the BPD cluster had a central position. Within the SUD symptom cluster, three pairs of symptoms (“tolerance” with “using longer than intended”; “tried to cut down or quit” with “keep using despite health or psychological problems”; and “time spent using or acquiring substance” with “reduced time on social/other activities”) were closely connected. This means that the two symptoms in each pair were strongly related, which is very much in line with previous network approaches to SUD symptoms in a community population [47], as well as in a clinical sample [48]. This may indicate that changes in some of the SUD symptoms during treatment may have a facilitating effect on changes in other symptoms, with an increase in the speed of recovery. It comes as no surprise that success in cutting down on one’s substance use (the most central symptom) may have direct positive effects on other aspects of an SUD [48].

The male and female symptom networks that we obtained were very similar in their centrality of hyperactivity symptoms. Similarly, in both networks SUD and BPD symptom clusters appeared unconnected to other categories. Although the symptom network for males looked denser, with a greater number of connections between symptoms and with stronger connections than for females, these differences did not reach statistical significance. The lack of significant gender differences in the symptom network is in line with inconsistencies in the literature regarding gender effects in relation to psychiatric comorbidity in patients with SUD [15,17,49,50,51].

It has been suggested that gender differences in comorbidity patterns for SUD vary by the main substance of abuse [52], and by the specific comorbid disorder [53]. For instance, among patients with MDD, suicidal ideation was related to illicit substance use and antisocial personality traits, while thoughts of worthlessness and guilt were related to alcohol and nicotine use. Furthermore, in males, psychomotor symptoms of depression (agitation/retardation) were more prominent, whereas in females, depressed mood, appetite/weight changes, and fatigue were more prominent [54]. Unfortunately, in the current study such detailed information on the different symptoms of MDD was not available, reducing the likelihood of finding gender effects on MDD within the symptom networks.

Sex or gender differences have been documented for all phases of substance use disorders [55]. For instance, men are twice as likely to have a lifetime SUD than women [56]. Men are more likely than women to use almost all types of illicit drugs [57], whilst their illicit drug use is more likely to result in emergency room visits or overdose deaths than that of women. In contrast, women more frequently use sedatives compared to men, report more cravings [58,59,60], and show higher relapse rates [61,62,63]. Furthermore, some studies suggest that females progress more rapidly than males through the drug usage stages [64]. Consequently, it has been argued that there is a need for a gender perspective in the treatment of, and research into, substance use disorders [65]. However, the focus of the present study on cross-disorder associations between symptoms makes it difficult to compare the results to previous research on sex/gender and SUD.

### 4.1. Limitations

The main limitation of the present study related to its cross-sectional design. This limited the degree to which potential causative associations between variables could be inferred. Also, our participants had to recall information from childhood retrospectively for some of the symptoms (i.e., ADHD and CD), adding a level of error in these historical data. Similarly, when employing rating scales, limitations arose in relation to the inherent subjective nature of certain symptoms, introducing recall bias as a possible source of unreliability [66,67]. Furthermore, complete information on all individual symptoms was only available for ADHD and BPD. For SUD, CD, and MDD, information was available only for a subset of symptoms, limiting the analyses to only a subset of the psychiatric disorders commonly observed in patients with SUD, and only some of their symptoms. The reason for this was that, as per the skip patterns on the instrument used, for respondents who were negative on the first few symptoms of ASP and MDD, the rest of the symptoms were skipped. In particular, the representation of MDD symptoms was limited, as only depressed mood (current and lifetime) data were available. We acknowledge the limitation of assessing only one or two symptoms of a complex condition like depression, and the potential impact of this on the building of the network model. However, it was valuable to include symptoms of MDD, as they have an important role and connections in the network. If they had been assessed more extensively, the connectivity of depressive symptoms with the other symptom nodes might have been more prominent. It should be noted that in the symptom network approach, it does not matter how many symptoms are assessed or whether all criteria of a certain condition are assessed. Furthermore, participants were recruited by convenience, and it is unclear how representative the samples may be either for adults with SUD seeking treatment, or in regard to the overall SUD population in the countries studied.

Additionally, the presence of SUD was assessed using DSM-IV criteria/symptoms; thus, our findings might not fully generalize to DSM-5 criteria. Most of the SUD criteria (10 out of 11) are the same in DSM-5, except for the criminalization criterion; also, one criterion was added—craving. Importantly, the 10 criteria that overlap between DSM-IV and DSM-5 are in fact the most relevant from a network point of view, given their connectedness. However, the position of the new DSM-5 “craving” criterion could not be addressed in the current study. In the transition from DSM-IV to DSM-5, a more dimensional approach was also introduced, where the severity of SUD is assessed by adding up the number of individual SUD symptoms. This further emphasizes the importance of examining individual symptoms and how these interact, as was carried out through the network analyses applied here. It must be noted that our sample consisted of SUD patients in specialized addiction care facilities. Previous studies suggest potential differences in DSM–SUD–symptom networks between substance users in the general population {Rhemtulla, 2016 #11257} and SUD patients in specialized addiction care facilities {Rutten, 2021 #11818}. Future studies might also explore potential differences in SUD-symptom networks between patients with mild, moderate, or severe SUD.

Furthermore, although our network analyses identified some “bridge symptoms”, the relevance of these remains to be studied. Directing future research on comorbidity toward identification of bridge symptoms, and incorporating novel quantitative methods such as those proposed by Jones et al. (2021) to identify bridge symptoms in network analyses are promising next steps [68]. Finally, it should be noted that low threshold correlations (<0.25) between nodes (symptoms) were not reported in our network diagram. Although this is a commonly applied and accepted method to visually reduce the number of edges, a more lenient threshold approach would have yielded a larger number of edges, and potentially may have identified more bridge symptoms. Therefore, replication of our results in large longitudinal or population-wide studies, using a broad assessment of psychiatric symptoms, is required to draw definitive conclusions about psychiatric symptom networks in patients with SUD, and the potential sex differences herein.

### 4.2. Clinical Implications and Future Research Directions

Despite the many criticisms, the current study corroborates the validity of the DSM classification of common mental disorders (including SUD), and its use in future studies on dual disorders. ASP/CD and ADHD share some symptoms that can be viewed as bridge symptoms and may represent a developmental course for some patients with ADHD who later develop ASP. Unexpectedly, no bridge symptoms were seen with SUD, and this needs to be explored further. In the treatment of patients with SUD, focusing on the central symptoms may be more efficient than working on the less central symptoms, and similarly, working on bridge symptoms may be efficacious in the treatment of comorbid disorders. Finally, the impact of sex and gender, and related variables, on SUD and its comorbidities needs further investigation.

Prospective network analysis (NA) studies that focus on changes in a broad range of symptoms either as a naturalistic follow-up study, or during a treatment study, would shed light on the developmental course and interactions of symptoms of comorbid disorders during the recovery process. Future NA research should include a broader range of symptoms (e.g., all DSM depression symptoms), additional comorbid disorders (e.g., anxiety disorders and posttraumatic stress disorder), other clinical features (i.e., executive functioning and personality variables), and/or include variables from other clinically relevant domains (e.g., response to treatment and quality of life).

## 5. Conclusions

We explored the network structure for symptoms of SUD, ADHD, CD, MDD, and BPD in adults seeking treatment for SUD, and we compared the symptom networks for males and females. Our network analyses identified seven clusters of symptoms, corresponding with the DSM diagnostic categories. There were few connections between symptom clusters; those that existed were mainly between some hyperactivity symptoms and CD and depressive symptoms. ADHD hyperactivity was most central in the network. We found no significant gender differences in the structure of the symptom networks. Our findings support the categorical DSM classification of disorders in treatment-seeking SUD patients with co-occurring mental disorders. Future studies should include a broader range of comorbid disorders and symptoms, and prospectively explore changes in the symptoms network of patients during treatment, as well as focus on other indicators such as treatment outcome and quality of life.

## Figures and Tables

**Table 1:** Legend of symptom descriptions to identify nodes.

Node	Disorder and Symptom Descriptions
	**Substance Use Disorder**
S1	tolerance
S2	abstinence or withdrawal
S3	used more or longer than intended
S4	tried to cut down or quit
S5	spent time using or acquiring substance
S6	reduced time on social or other activities for substance use
S7	keep using despite health or psychological problems
	**Inattention**
I1	fails to give close attention/make careless mistakes
I2	difficulty sustaining attention
I3	does not seem to listen when spoken to directly
I4	Difficult to follow through on instructions/fails to finish projects
I5	difficulty organizing tasks or activities
I6	avoid, dislike, reluctant to tasks that require sustained mental effort
I7	often loses things necessary for tasks or activities
I8	often easily distracted by extraneous stimuli
I9	often forgetful in daily activities
	**Hyperactivity/Impulsivity**
H1	fidgets a lot when seated
H2	often trouble remaining seated
H3	often experience feelings of restlessness
H4	difficulty being as quiet as others
H5	always on the go
H6	often talks too much
H7	often answer questions before completed
H8	often have trouble waiting for your turn
H9	often interrupts others
	**Conduct Disorder**
C1	skipped school or stayed out < age 15
C2	Lie cheat steal < age 15
C3	Fights, threatened, intimidated others < age 15
C4	destroyed others property or set fires < age 15
C5	maltreatment animals or cruelty to people < age 15
	**Borderline Personality Disorder**
B1	you got upset at the thought of someone leaving you
B2	ups and downs in relationships with people you care about
B3	feeling of who you are and which direction you are going has suddenly changed
B4	often done things impulsively
B5	tried to injure yourself or threatened to do so
B6	many sudden mood swings
B7	you often feel empty inside
B8	often angry outbursts or so angry that you lose control
B9	when under great stress, feeling suspicious or alienated towards people
	**Major Depression**
D1	depressed mood (current)
D2	depressed mood (lifetime)

**Table 2:** Demographic and clinical characteristics of SUD treatment-seeking participants, by gender (*n* = 772).

	Males	Females	Total Sample ^a^
	*n* (%)	*n* (%)	*n* (%)
	597 (77.33)	175 (22.67)	772 (100)
Age Mean *(SD)*	37.99 (11.23)	36.86 (10.98)	37.74 (11.17)
Ethnicity **			
Caucasian	351 (64.17)	108 (72.97)	459 (66.04)
Hispanic	175 (31.99)	29 (19.59)	204 (29.35)
Other	21 (3.84)	11 (7.43)	32 (4.60)
Marital status			
Single/Divorced	458 (78.16)	123 (71.93)	581 (76.75)
Housing			
Homeless/In shelter	105 (18.45)	32 (19.16)	137 (18.61)
Alone	185 (32.51)	61 (36.53)	246 (33.42)
With Partner/Friends/Parents	279 (49.03)	74 (44.31)	353 (47.96)
Employment *			
Employed	192 (33.10)	39 (23.35)	231 (30.92)
Unemployed	299 (51.55)	94 (56.29)	393 (52.61)
Sick leave/Disability	89 (15.34	34 (20.36)	123 (16.47)
Main substance used *			
Alcohol	220 (37.23)	61 (35.26)	281 (36.78)
Amphetamines	35 (5.92)	17 (9.83)	52 (6.81)
Cannabis	123 (20.81)	20 (11.56)	143 (18.72)
Cocaine	72 (12.18)	17 (9.83)	89 (11.65)
Heroin	90 (15.23)	31 (17.92)	121 (15.84)
Prescription opioids	20 (3.38)	12 (6.94)	32 (4.19)
Methadone	12 (2.03)	6 (3.47)	18 (2.36)
Other	19 (3.21)	9 (5.20)	28 (3.66)
Psychiatric comorbidity			
Adult ADHD *	85 (14.60)	15 (8.72)	100 (13.26)
Conduct disorder (<age16)	159 (46.09)	52 (48.15)	211 (46.58)
ASP	122 (35.67)	48 (45.28)	170 (37.95)
BPD ***	131 (21.94)	62(35.43)	193 (25.00)
MDD	152 (48.56)	56 (56.57)	208 (50.49)

^a^ Total *n* varies because of missing data points. ADHD = Attention Deficit/Hyperactivity Disorder; ASP = Antisocial personality disorder; BPD = Borderline personality disorder; MDD = Major depressive disorder, lifetime; * *p* < 0.05; **p < 0.01; *** *p* < 0.001.

## Data Availability

The data presented in this study are available from the authors upon request.

## Supplementary Materials

The following are available online at https://www.mdpi.com/article/10.3390/jcm11102883/s1, Figure S1: Three centrality measures of the nodes in the network of 41 DSM symptoms of SUD, ADHD, CD, MDD, and BPD for entire sample (*n* = 772); Figure S2: Three centrality measures of the nodes in the network of 41 DSM symptoms of SUD, ADHD, CD, MDD, and BPD compared by gender; Table S1: Estimated thresholds for 41 DSM symptoms of SUD, ADHD, CD, MDD, and BPD for the entire sample (*n* = 772), and the subsamples of males (*n* = 597) and females (*n* = 175).
