# Supplementary material for: Network Analysis of DSM Symptoms of Substance Use Disorders and Frequently Co-Occurring Mental Disorders in Patients with Substance Use Disorder Who Seek Treatment
Source: J Clin Med. 2022 May 19;11(10):2883. doi: 10.3390/jcm11102883 (PMC9145186; doi:10.3390/jcm11102883)
Supplement: Supplementary file 1 [file jcm-11-02883-s001.zip › jcm-1700767-supplementary.pdf]

Journal of
Clinical Medicine

MDPI

Network analysis of DSM symptoms of substance use disorders and frequently co-occurring mental disorders in patients with substance use disorder who seek treatment

Table S1 Estimated thresholds for 41 DSM symptoms of SUD, ADHD, CD, MDD, and BPD for the entire sample (n = 772) and the subsamples of males (n = 597) and females (n = 175). Symptoms with higher thresholds are more likely to occur in the population sampled

	Thresholds		
**Symptom**	**Total sample** (n = 772)	**Males** (n = 597)	**Females** (n = 175)
I1	−2.56	−2.08	−2.82
I2	−2.95	−2.81	−1.49
I3	−1.77	−2.16	−1.37
I4	−1.96	−2.16	−1.76
I5	−2.33	−2.13	−1.94
I6	−1.75	−1.68	−1.70
I7	−1.49	−1.45	−1.36
I8	−1.98	−1.70	−1.28
I9	−2.09	−1.86	−1.55
H1	−1.82	−1.25	−1.77
H2	−2.52	−2.66	−1.12
H3	−2.24	−2.31	−0.84
H4	−2.90	−2.68	−2.00
H5	−1.69	−1.64	−1.21
H6	−2.04	−2.34	−1.64
H7	−1.75	−1.68	−1.21
H8	−1.88	−1.51	−0.95
H9	−3.15	−2.96	−2.43
B1	−2.25	−2.28	−0.97
B2	−2.15	−2.08	−1.45
B3	−2.24	−2.06	−2.01
B4	−0.52	−0.52	−0.20
B5	−2.15	−2.06	−0.99
B6	−2.83	−2.85	−0.95
B7	−1.63	−1.60	−1.16
B8	−2.40	−2.28	−1.58
B9	−2.21	−2.29	−1.59
D1	−1.96	−2.37	−0.86
D2	−0.05	0.03	0.67
C1	−1.45	−1.31	−0.91
C2	−1.51	−1.25	−1.37
C3	−1.93	−1.89	−1.31
C4	−2.36	−2.54	−1.76
C5	−3.37	−2.82	−2.96
S1	−1.65	−1.49	−1.08
S2	−1.36	−0.99	−2.03
S3	−1.41	−0.93	−1.25
S4	−2.67	−2.60	−1.44
S5	−1.23	−1.26	−1.10
S6	−2.11	−2.02	−1.73
S7	−1.24	−1.11	−0.77

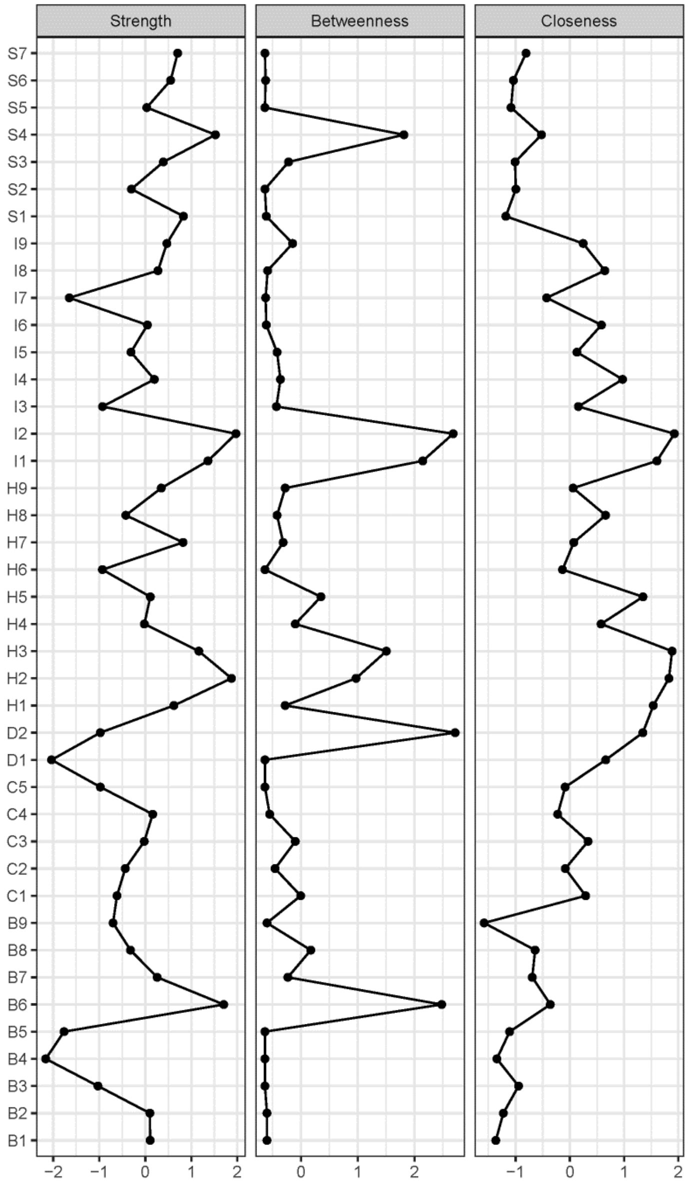

Figure S1. Three centrality measures of the nodes in the network of 41 DSM symptoms of SUD, ADHD, CD, MDD, and BPD for entire sample (*n*=772). From left to right: node strength, betweenness, and closeness. Standardized *z*-scores are shown on the x-axis.

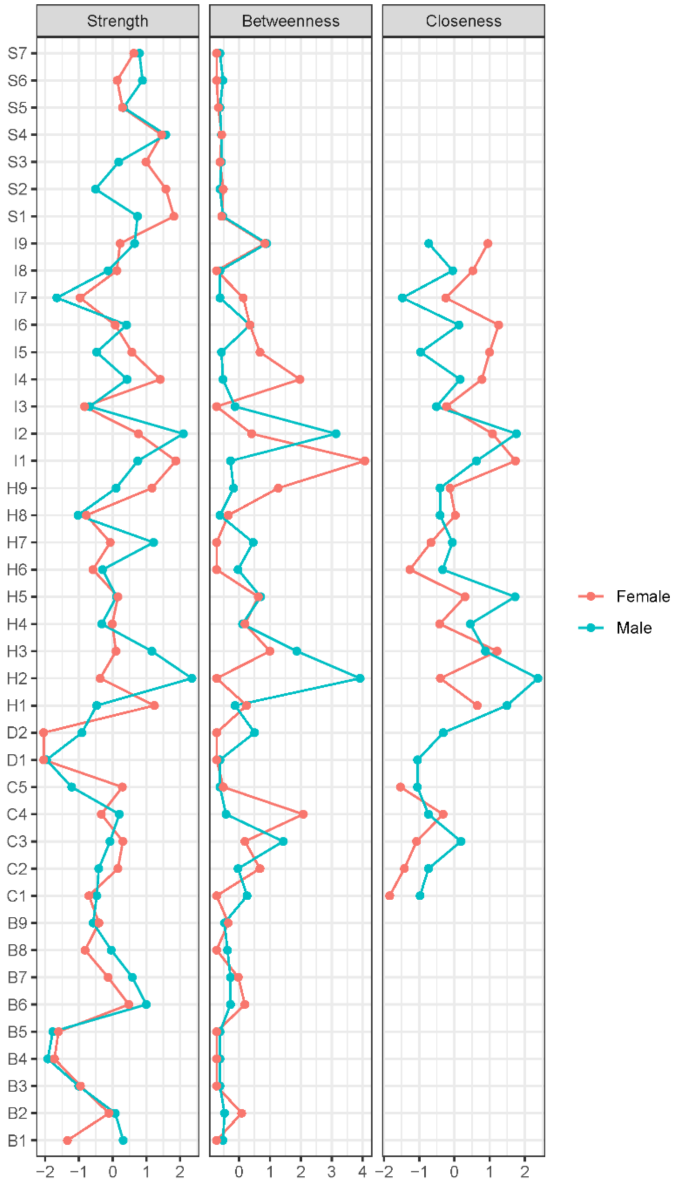

Figure S2. Three centrality measures of the nodes in the network of 41 DSM symptoms of SUD, ADHD, CD, MDD, and BPD, compared by gender. From left to right: node strength, betweenness, and closeness. Standardized *z*-scores are shown on the x-axis.
