# Supplementary material for: Network Analysis of DSM Symptoms of Substance Use Disorders and Frequently Co-Occurring Mental Disorders in Patients with Substance Use Disorder Who Seek Treatment
Source: J Clin Med. 2022 May 19;11(10):2883. doi: 10.3390/jcm11102883 (PMC9145186; doi:10.3390/jcm11102883)
Supplement: Supplementary file 1 [file jcm-11-02883-s001.zip › jcm-1700767-supplementary-ICASA group members.pdf]

ICASA network group members

Josep Antoni Ramos-Quiroga, MD, PhD
Department of Psychiatry, Hospital Universitari Vall d'Hebron, CIBERSAM
Group of Psychiatry, Mental Health and Addiction, Vall d'Hebron Research Institute
Department of Psychiatry and Forensic Medicine, Universitat Autònoma de Barcelona
Barcelona, Catalonia, Spain

Matthijs Blankers, PhD
Arkin Mental Health Care, Department of Research, Amsterdam, the Netherlands;
Trimbos institute, Netherlands Institute of Mental Health and Addiction, Utrecht, the Netherlands;
Amsterdam UMC, University of Amsterdam, Department of Psychiatry, Amsterdam, The Netherlands

Lara Grau, MD, PhD
Department of Psychiatry, Hospital Universitari Vall d'Hebron, CIBERSAM
Associated Professor of Psychiatry, Universitat Autònoma de Barcelona
Barcelona, Catalonia, Spain

Mathias Luderer, MD, PhD
Dpt. of Psychiatry, Psychosomatic Medicine and Psychotherapy, University Hospital, Goethe University Frankfurt, Germany

Frances R. Levin, MD
Columbia University Medical Center, New York State Psychiatric Institute, New York, NY
United States of America

Sharlene Kaye, PhD
Research Unit, Justice Health and Forensic Mental Health Network
National Drug and Alcohol Research Centre, University of New South Wales
Sydney, Australia

Prof. Zsolt Demetrovics
Centre of Excellence in Responsible Gaming, University of Gibraltar, Gibraltar
Addiction Research Group, ELTE Eötvös Loránd University, Budapest, Hungary

Geurt van de Glind, PhD
Utrecht University of Applied Sciences, Utrecht, The Netherlands

Prof. Franz Moggi, PhD, EMBA
University Hospital of Psychiatry and Psychotherapy, University of Bern, Bern
Switzerland

Maija Konstenius, PhD
Section Head Outpatient Care, Stockholm Centre for Dependency Disorders
Dept of Clinical Neuroscience, Karolinska Institutet
Sweden

Geert Dom, MD PhD
Antwerp University (UA, CAPRI), Belgium
Psychiatric Center Multiversum, Boechout, Belgium

Frieda Matthys, MD PhD
Department of Psychiatry, University Hospital Brussels, Vrije Universiteit Brussel (VUB)
Belgium

Máté Kapitány-Fövény, PhD
Semmelweis University Faculty of Health Sciences, Budapest, Hungary.
Nyírő Gyula National Institute of Psychiatry and Addictions, Budapest,
Hungary

Cleo L. Crunelle, PhD, PhD
Department of Psychiatry, University Hospital Brussels (UZ Jette)
Toxicological Center, University of Antwerp
Belgium

Johan Franck, MD PhD
Stockholm Centre for Dependency Disorders
Dept of Clinical Neuroscience, Karolinska Institutet
Sweden

Romain Icick, MD, PhD
CSAPA Espace Murger
Hôpital Fernand Widal
France

Alex Begeman, MA
De Hoop ggz, Department Education and Research, Clinic VZ ZW
The Netherlands

Christoffer Brynte, MD
Dept of Clinical Neuroscience, Karolinska Institutet
Sweden

Sofie Verspreet, MA
Multiversum, Mental Health care

Belgium

Michiel van Kernebeek, MD
University Hospital Brussels (UZ Jette)
Belgium

Constanza Daigre Blanco, PhD
Department of Psychiatry, Hospital Universitari Vall d'Hebron
Spain

Regina Sala, MD, PhD
Centre for Psychiatry, Wolfson Institute
Barts & The London School of Medicine & Dentistry
Queen Mary University of London
United Kingdom

Martin Holtmann, MD
Chair of Child and Adolescent Psychiatry, Ruhr-University Bochum
Medical Director, LWL-University Hospital for Child and Adolescent Psychiatry, Hamm
Germany

Moritz Noack, MD
LWL-University Hospital for Child and Adolescent Psychiatry, Hamm
Germany

Csaba Barta, MD, PhD
Institute of Medical Chemistry, Molecular Biology and Pathobiochemistry, Semmelweis University, Budapest, Hungary
Pázmány Péter Catholic University, Faculty of Humanities and Social Sciences, Institute of Psychology, Budapest, Hungary

Natalie C. Sánchez-García, PhD
PhD Program in Clinical Psychology, Universidad Carlos Albizu, San Juan, Puerto Rico
School of Behavioral and Brain Sciences, Ponce Health Sciences University, Ponce, Puerto Rico

Steve Faraone, PhD
Institute for Human Performance (IHP)
Upstate Medical University, Syracuse, NY

Marta Ribasés
VHIR Vall d'Hebron Research Institute
Psychiatry, Mental Health and Addictions Research Group
Barcelona, Catalonia, Spain

Renske Spijkerman
Parnassia Addiction Research Center (PARC)
Nijmegen, The Netherlands

Ilse Truter
Distinguished Professor
Leader of DURU
Director, School of Clinical Care & Medicinal Sciences
Drug Utilization Research Unit, Department of Pharmacy,
Faculty of Health Sciences
Nelson Mandela University
Port Elizabeth, South Africa

Corné Coetzee
Senior Lecturer Pharmacology
Department of Pharmacy
University of Limpopo
Polokwane, South Africa

Izabela Przezdzik
Department of Cognitive Neuroscience,
Radboud University Medical Centre,
Nijmegen, The Netherlands

Amy Yule
Boston Medical Center,
Boston University School of Medicine, USA

Florence Vorspan
Paris, France
